# Nocturnal Glucose Profile According to Timing of Dinner Rapid Insulin and Basal and Rapid Insulin Type: An *Insulclock^®^* Connected Insulin Cap-Based Real-World Study

**DOI:** 10.3390/biomedicines12071600

**Published:** 2024-07-18

**Authors:** Fernando Gómez-Peralta, Xoan Valledor, Cristina Abreu, Elsa Fernández-Rubio, Laura Cotovad, Pedro Pujante, Sharona Azriel, Jesús Pérez-González, Alba Vallejo, Luis Ruiz-Valdepeñas, Rosa Corcoy

**Affiliations:** 1 Endocrinology and Nutrition Unit, Hospital General de Segovia, Luis Erik Clavería Neurólogo S.N Street, 40002 Segovia, Spain; cabreupadin@gmail.com; 2 Research and Development Unit, Insulcloud S.L., 28020 Madrid, Spain; xoan@insulcloud.com (X.V.); suso@insulclock.com (J.P.-G.); alba@insulcloud.com (A.V.); luis@insulcloud.com (L.R.-V.); 3 Endocrinology and Nutrition Service, Cruces University Hospital, 48903 Barakaldo, Spain; elsa.fernandezrubio@osakidetza.eus; 4 Endocrinology and Nutrition Service, Hospital Arquitecto Marcide, 15405 Ferrol, Spain; lauracotovad@hotmail.com; 5 Endocrinology and Nutrition Service, Hospital Universitario Central de Asturias, 33011 Oviedo, Spain; pedropujanteal@gmail.com; 6 Endocrinology and Nutrition Service, Hospital Universitario Infanta Sofía, 28702 San Sebastián De Los Reyes, Spain; sharona.azriel@gmail.com; 7 Endocrinology and Nutrition Service, Hospital de la Santa Creu i Sant Pau, Institut de Recerca, 08041 Barcelona, Spain; rcorcoy@santpau.cat; 8 Departament de Medicina, Universitat Autònoma de Barcelona, 08193 Barcelona, Spain; 9 CIBER-BBN, 28029 Madrid, Spain

**Keywords:** ultrarapid insulin, second-generation basal insulin, nocturnal hyperglycemia, nocturnal hypoglycemia, connected insulin pen cap

## Abstract

Background: A study to assess the glucose levels of people with type 1 diabetes (T1D) overnight, based on the insulin type and timing. Methods: A real-world, retrospective study of T1D, using multiple daily insulin injections. Continuous glucose monitoring and insulin injection data were collected for ten hours after dinner using the *Insulclock^®^* connected cap. Meal events were identified using the ROC detection methodology. The timing of the rapid insulin, second injections, and the type of insulin analogs used, were evaluated. Results: The nocturnal profiles (n = 775, 49 subjects) were analyzed. A higher glucose AUC of over 180 mg/dL was observed in subjects with delayed injections (number; %; mg/dL × h): −45–15 min (n = 136; 17.5%, 175.9 ± 271.0); −15–0 min (n = 231; 29.8%, 164.0 ± 2 37.1); 0 + 45 min (n = 408; 52.6%, 203.6 ± 260.9), (*p* = 0.049). The use of ultrarapid insulin (FiAsp^®^) (URI) vs. rapid insulin (RI) analogs was associated with less hypoglycemia events (7.1 vs. 13.6%; *p* = 0.005) and TBR70 (1.7 ± 6.9 vs. 4.6 ± 13.9%; *p* = 0.003). Users of glargine U300 vs. degludec had a higher TIR (70.7 vs. 58.5%) (adjusted R-squared: 0.22, *p* < 0.001). The use of a correction injection (n = 144, 18.6%) was associated with a higher number of hypoglycemia events (18.1 vs. 9.5%; *p* = 0.003), TBR70 (5.5 ± 14.2 vs. 3.0 ± 11.1%; *p* = 0.003), a glucose AUC of over 180 mg/dL (226.1 ± 257.8 vs. 178.0 ± 255.3 mg/dL × h; *p* = 0.001), and a lower TIR (56.0 ± 27.4 vs. 62.7 ± 29.6 mg/dL × h; *p* = 0.004). Conclusion: The dinner rapid insulin timing, insulin type, and the use of correction injections affect the nocturnal glucose profile in T1D.

## 1. Introduction

Maintaining a safe and stable blood glucose level during the night is crucial for people with type 1 diabetes (T1D). Determining the appropriate pre-dinner rapid insulin and basal insulin to adequately control both postprandial and nocturnal glucose can be a daily challenge, with a high level of uncertainty. Many factors, which are difficult to measure and predict, can affect the nocturnal glycemic profile, one of them being exercise during the day [1]. The safety and quality of life of individuals with T1D are clearly impacted by these factors [2].

Insulin regimens requiring multiple daily injections (MDIs) impose a considerable burden on people with T1D [3]. Previous studies have shown that the timing of prandial insulin injection can affect postprandial glucose levels [4,5]. However, these studies were conducted in laboratory settings or relied on self-reported insulin injection times and doses. Notably, a large percentage of people with T1D do not follow the recommended prandial insulin injection timing, which can result in different glucose dynamics and increase both postprandial excursions and nocturnal hypoglycemia risk [6].

Manufacturers recommend injecting regular human insulin 30–45 min before starting a meal, 15 min in the case of rapid insulin (RI) analogs, and at the start of a meal or within 20 min for second-generation (“ultrarapid”) insulin (URI) [7,8]. There is limited scientific evidence on the nocturnal glucose profile depending on the timing and type of pre-dinner rapid insulin injections. However, connected insulin pens and caps can now automatically track continuous glucose monitoring (CGM) data, as well as the dose and exact time of insulin injections [9]. This is a promising development, as it can help people with T1D to manage their blood sugar levels more effectively and, ultimately, reduce the risk of complications [10].

Insulin therapy adherence can be influenced by socioeconomic factors, treatment complexity, and fear of hypoglycemia [11,12]. Errors in insulin administration, such as bolus omissions and delays, can prevent optimal glycemic control [13]. This can negatively impact the quality of life of people with diabetes and increase the risk of morbidity, mortality, and hospitalization [14].

Although it is recommended to administer the prandial bolus injection at least 15 min before mealtimes [15], it may not always be feasible in real life. Fortunately, URI is available, which can improve postprandial dynamics, even when injected after the meal has started. Our previous study, which focused on postprandial glucose dynamics, confirmed a reduction in both immediate hyper- and late hypoglycemia by using URI in a real-life setting [6].

Second-generation basal insulin (BI) analogs have been developed to help people with T1D face daily challenges. These analogs, such as insulin glargine 300 U/mL (Gla-300) and insulin degludec (IDeg), have a longer and flatter profile, with less variability [16]. However, randomized clinical trials have not consistently detected significant differences between the two insulins, using the CGM methodology [17]. In an MDI regimen, the different effects on the glucose profile caused by the two basal insulins are more easily observed during the night [18].

People with diabetes often administer additional (correction) insulin doses to immediately offset postprandial peaks [19]. Excessive correction frequently leads to postprandial hypoglycemia due to the “stacking insulin effect” [20].

*Insulclock^®^* is an innovative small cap designed to be easily attached to disposable insulin pens. Its primary function is to accurately record crucial information, such as the date, time, duration, and dose of insulin injections [21]. This recorded data is seamlessly integrated with other pertinent health metrics, including glucose levels from CGM devices or glucometers, dietary intake, and physical activity, through the user-friendly *Insulclock^®^* app. Patients are able to access and review the comprehensive data collected, enabling them to effectively monitor their health trends and patterns. Moreover, this information can be securely shared with their healthcare providers, facilitating collaborative analysis and personalized treatment plans. Notably, a multicenter randomized controlled trial has demonstrated the positive impact of the system on glycemic control and variability, adherence to insulin treatment, and overall quality of life for individuals with T1D and inadequate control [10].

The current study aimed to analyze the nocturnal glucose profile in people with T1D using MDIs, according to the timing of dinner rapid insulin and the type of rapid and basal insulin used.

## 2. Materials and Methods

### 2.1. Design

A retrospective study was carried out using anonymous, real-world data from the *Insulclock^®^* electronic database, from six participating centers in Spain. These centers were the Hospital General de Segovia in Segovia, Cruces University Hospital in Barakaldo, Hospital Arquitecto Marcide in Ferrol (A Coruña), Hospital Universitario Central de Asturias in Oviedo, Hospital Universitario 12 de Octubre in Madrid, and Hospital Universitario Infanta Sofía in San Sebastián de los Reyes.

At the beginning of the *Insulclock^®^* use, all participants provided written informed consent, allowing Insulcloud S.L. to collect and use their anonymized and tabulated data for scientific purposes. The study adhered to the ethical principles in the Declaration of Helsinki and was approved by the Research Ethics Committee of the Hospital General de Segovia in Segovia, Spain, before any study-related activities were undertaken.

### 2.2. Population and Database

The study analyzed data from consecutive T1D participants, who started using the *Insulclock^®^* connected insulin pen cap [21] from January to June 2022. The type of insulin used was not an inclusion criterion. The analysis focused on overnight periods, consisting of ten hours starting at dinner time, and included data from continuous glucose monitoring (CGM) and insulin injections. Only dinner glycemic excursions, starting with a glucose level between 70 mg/dL (3.9 mmol/L) and 250 mg/dL (13.9 mmol/L), and with 10 h data after dinner initiation, were included in the analysis.

The Glucose Rate Increase Detector (GRID) algorithm was employed to identify dinner times, by analyzing excursions in glucose levels between 19 to 23:59 h [22]. The algorithm estimates the rate of change in glucose levels from CGM data. It identifies glucose excursions by looking for a gradient ≥ 95.4 mg/dL/h (5.3 mmol/L/h) for two consecutive readings within 30 min, or ≥90 mg/dL/h (5.0 mmol/L/h) for three consecutive readings within 45 min, when the CGM signal is >129.6 mg/dL/h (7.2 mmol/L). The GRID algorithm has high specificity to detect meal-related glucose excursions and has been clinically validated for use with Automated Insulin Delivery (AID) systems and MDIs [23].

It is worth noting that all patients had been previously using CGM (*Freestyle Libre2^®^*), as part of their usual diabetes care.

### 2.3. Outcomes

This study assessed the impact of the rapid insulin injection timing, by comparing three groups of nocturnal glucose profiles based on when the injection was administered in relation to the start of the post-dinner rise in glucose levels (PE). The groups were: injections 45 to 15 min before (−45/−15), injections within 15 min before the PE onset (−15/0), and injections given from the start of the rise to 45 min after (0/+45).

The glucometrics and thresholds used to describe the glycemic dynamics during the analyzed nighttime periods were in line with the recommendations in the “Continuous glucose monitoring and metrics for clinical trials: an international consensus statement” [24].

To assess the magnitude of overnight hyperglycemia, we conducted a calculation of the area under the curve (AUC) for glucose levels surpassing the recommended upper limit of 180 mg/dL (10 mmol/L).

Hypoglycemic events are defined as periods when glucose levels are below 70 mg/dL (3.9 mmol/L) for more than 15 min. Two variables are used to quantify hypoglycemia: the percentage of overnight periods with a hypoglycemic event, and the time spent below the range of 70 mg/dL (3.9 mmol/L) (TBR70) in regard to glucose.

The time spent within the recommended target range of 70–180 mg/dL (3.9–10.0 mmol/L) (TIR) during the nighttime period was also assessed.

The differences in nocturnal glycemic profiles, according to the insulin type used, were evaluated. The study compared the two second-generation BI analogs, glargine 300 U/mL vs. insulin degludec, and the first-generation rapid insulin analogs (lispro, aspart, and glulisine) (RI) vs. second-generation “ultrarapid” insulin (URI) analogs (Fiasp^®^, Novo Nordisk, Denmark).

Additionally, the administration of a second injection (correction dose) 1–5 h after the first rapid insulin dose at dinner was also evaluated.

### 2.4. Statistical Analyses

Statistical analyses were performed using SPSS software, version 25.0 (Chicago, IL, USA). The level of statistical significance was set at a bilateral *p* < 0.05. Continuous variables were described by the mean and standard deviation (SD), when normally distributed, or by the median, interquartile range (IQR), when not normally distributed. Categorical variables were described by the number of valid cases and percentages. Comparisons of the proportions and/or frequency distributions were performed with the Chi-square test, Mann–Whitney, Kruskal–Wallis, or the ANOVA test, as appropriate, with the post-hoc Bonferroni correction.

Logistic and linear regression models were used to assess predictors of events of glucose under 70 mg/dL (3.9 mmol/L), time below the range of glucose 70 mg/dL (3.9 mmol/L) (TBR70), glucose AUC over 180 mg/dL, and time in range 70–180 mg/dL (3.9–10.0 mmol/L) (TIR), depending on timing of injection, use of a second injection, rapid and basal insulin type and pre-dinner glucose level. Simple regression models were first performed, and those variables reaching statistical significance were included in the multivariable regression models (forward selection). In the multivariable model, a *p*-value < 0.05 was considered significant. Those variables with a variance inflation factor >5 were removed from the models.

## 3. Results

### 3.1. Population

A total of 775 night periods were included, for 49 participants, 45.51 *±* 13.2 years old, 28 of whom were women (57.1%).

Table 1 summarizes the clinical characteristics and baseline glucometrics of the included population, both overall and according to the type of rapid and basal insulin used.

### 3.2. Nocturnal Glucose Dynamics Depending on the Rapid Insulin Injection Timing

The distribution of the analyzed night times, according to the dinner rapid insulin injection times, was as follows: −45/−15 injections accounted for 17.5% (n *=* 136), −15/0 injections for 29.8% (n *=* 231), and 0/+45 injections for 52.6% (n *=* 408).

**Figure 1:**
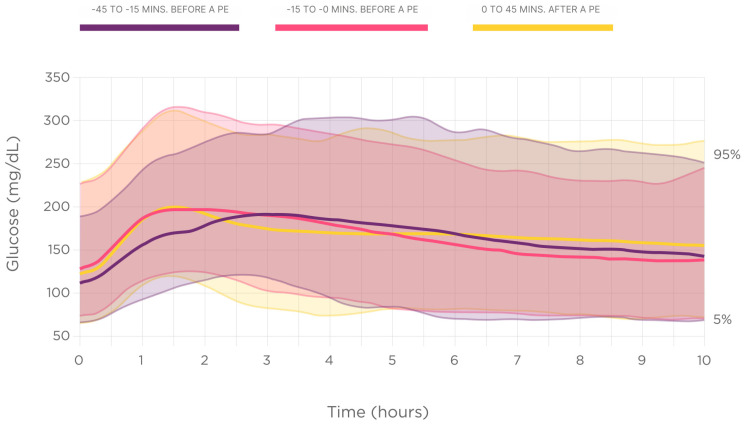
Nighttime glucose dynamics depending on the rapid insulin injection time.

Figure 1 displays the nocturnal glucose dynamics, depending on the dinner prandial insulin injection time.

**Figure 2:**
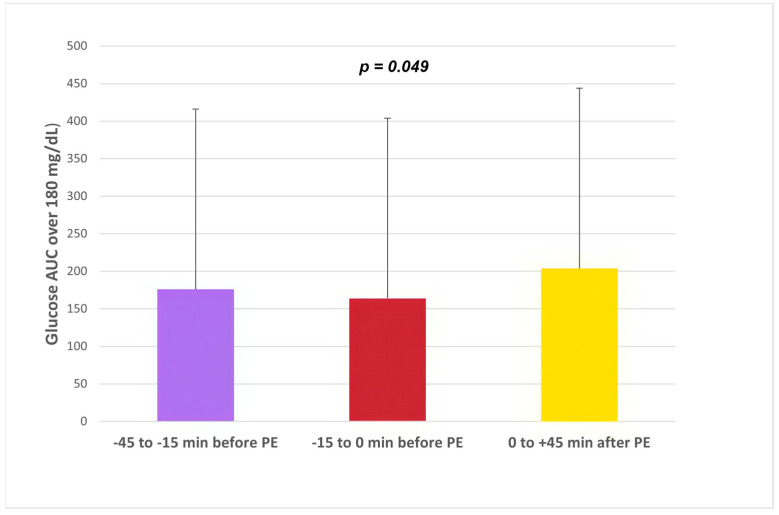
Nighttime glucose AUC of over 180 mg/dL [10 mmol/L] depending on the rapid insulin injection time.

The nocturnal high glucose excursion (glucose AUC of over 180 mg/dL [10 mmol/L]) results showed statistically significant differences between the groups, being higher in subjects with delayed rapid insulin injections 0/+45 min, (number, %, mg/dL × h): –45–15 min (n = 136, 17.5%, 175.96 ± 271.0); −15–0 min (n = 231, 29.8%,164.0 ± 237.1); 0+45 min (n = 408, 52.6%, 203.6 ± 260.9); overall *p =* 0.049 (Figure 2).

The rate of nocturnal hypoglycemic events did not differ according to the timing of the rapid insulin injection, mean ± SD: –45–15 min, 11.0 ± 0.3; –15–0 min, 9.5 ± 0.3; 0 + 45 min, 12.0 + 0.3 (*p =* 0.630).

The time below the glucose range of 70 mg/dL (3.9 mmol/L) (TBR70) showed similar results (mean ± SD): −45–15 min, 4.2 ± 13.7%; −15–0 min, 3.0 ± 10.7%; 0 + 45 min, 3.4 ± 11.7% (*p =* 0.674).

### 3.3. Analysis According to Dinner Ultrarapid Insulin Use

The nocturnal glucose AUC of over 180 mg/dL and TIR 70–180 did not reach statistically significant differences between URI vs. RI use (mean ± SD): 175.3 ± 248 vs. 204 ± 266 mg/dL × h (*p =* 0.366) and 61.7 ± 28.3 vs. 61.1 ± 30.8 mg/dL × h (*p =* 0.968).

**Figure 3:**
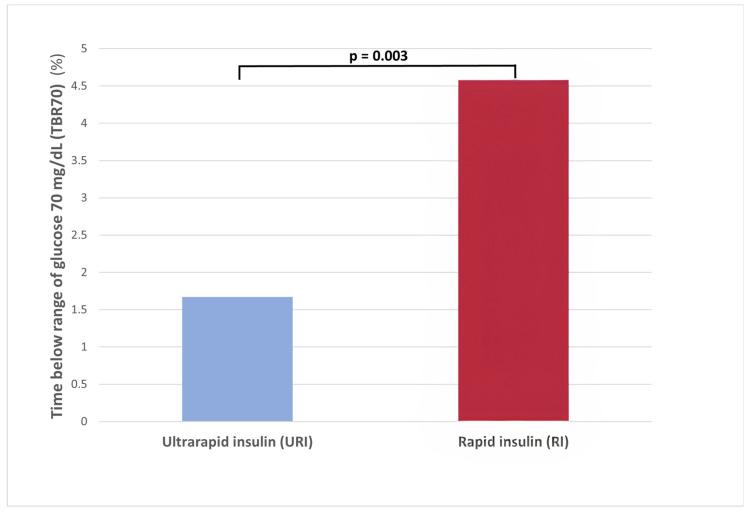
Time below the range of glucose 70 mg/dL (3.9 mmol/L) (TBR70) according to the use of ultrarapid insulin (URI) vs. rapid insulin (RI) analogs.

The use of URI vs. RI was associated with a lower TBR70 (1.7 ± 6.9 vs. 4.6 ± 13.9%; *p =* 0.003) (Figure 3) and less hypoglycemia events (7.1 vs. 13.6; *p =* 0.005) (Supplementary Figure S1).

### 3.4. Analysis According to the Second-Generation Basal Insulin Type

**Figure 4:**
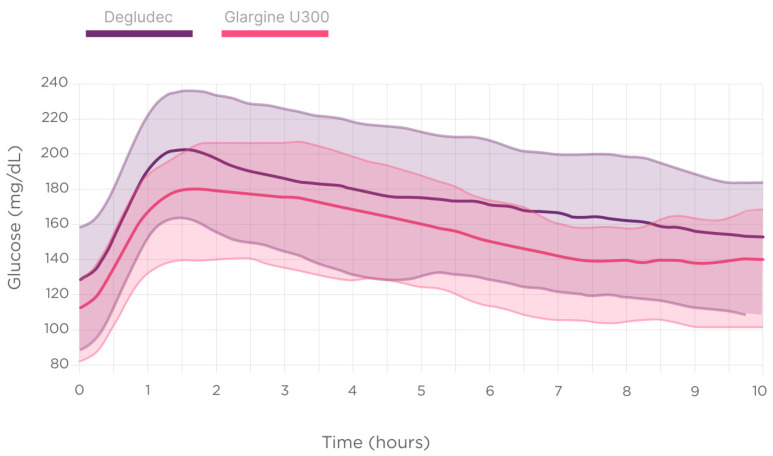
Nighttime glucose dynamics depending on the second-generation basal insulin type used (degludec vs. glargine U300).

Figure 4 describes the post-dinner and nocturnal glucose dynamics depending on the second-generation BI type.

Statistically significant differences were not detected regarding the type of second-generation BI used on the unadjusted glucometrics describing the nocturnal high glucose excursions (glucose AUC of over 180 mg/dL [10 mmol/L]), nocturnal hypoglycemic events, the time below the range of 70 mg/dL (3.9 mmol/L) (TBR70) in regard to glucose, nor the time in range 70–180 mg/dL (3.9–10.0 mmol/L) (TIR).

However, the multivariable logistic regression model, which included the timing of the injection, the BI type, and the pre-dinner glucose level, identified the use of Gla-300 as independently associated with a higher TIR 70–180 during the night (adjusted R-squared: 0.22, *p* < 0.001).

### 3.5. Analysis According to the Use of a Second Insulin Injection (Correction Dose)

A second (correction) injection 1–5 h after the first rapid insulin dose at dinner was detected in 18.6% of the analyzed overnight periods (n = 144). This practice was associated with a higher number of hypoglycemia events (18.1 vs. 9.5%; *p* = 0.003), TBR70 (5.5 ± 14.2 vs. 3.0 ± 11.1%; *p* = 0.003), a glucose AUC of over 180 mg/dL (226.1 ± 257.8 vs. 178.0 ± 255.3 mg/dL × h; *p* = 0.001), and a lower TIR (56.0 ± 27.4 vs. 62.7 ± 29.6 mg/dL × h; *p* = 0.004).

### 3.6. Multivariable Analysis

The multivariable logistic regression model (Supplementary Table S1), which included the timing of the injection, the use of a second injection, and the rapid and basal insulin type, confirmed the following:

- The use of a URI was independently associated with a reduced risk (−52%) of glucose events < 70 mg/dL (3.9 mmol/L) (adjusted R-squared: 0.017; *p* = 0.003), and with a lower (−64%) TBR70 (adjusted R-squared: 0.016; *p* = 0.003);
- Not adding a second (correction) injection after dinner was independently associated with:
  ○ A reduction in overnight hypoglycemia: −47% glucose events < 70 mg/dL), adjusted R-squared: 0.017, *p* = 0.003, and −61% TBR70 (adjusted R-squared: 0.016; *p* = 0.003);
  ○ A reduction in overnight hyperglycemia: −21% glucose AUC of over 180 mg/dL (adjusted R-squared: 0.017; *p* < 0.001);
  ○ More time in the recommended glucose range 70–180 mg/dL (TIR): +6.7% (62.7 ± 29.6 mg/dL vs. 56.0 ± 27.4) (adjusted R-squared: 0.048; *p* < 0.001).

Additionally, using glargine U300 instead of degludec was associated with a higher TIR (70.7 vs. 58.5%, +12.2%) after adjustment according to the baseline glucose before dinner (adjusted R-squared: 0.22; *p* < 0.001).

## 4. Discussion

Individuals diagnosed with T1D are advised to maintain a stable and safe blood glucose level during nocturnal hours, striving to avoid both hyperglycemia and hypoglycemia. The timing of prandial pre-dinner rapid insulin administration and the use of an ‘ultrarapid’ insulin analog have been identified in our study as factors contributing to improved glucose control overnight. Additionally, our findings suggest that the use of Gla-300, a second-generation basal insulin, as opposed to degludec, is associated with a greater likelihood of achieving the recommended glucose levels during the nocturnal period.

Several studies have been conducted to determine the effect of prandial insulin timing on postprandial glucose dynamics [4,15]. However, less scientific evidence is available evaluating the relationship between the recommended timing for pre-dinner prandial insulin injections and the nocturnal glucose profile beyond the postprandial period. A study comparing preprandial vs. postprandial insulin glulisine in patients initiating a basal–bolus regimen for type 2 diabetes showed that nocturnal hypoglycemia rates were higher in the postprandial administration group [19]. Another randomized, open-labeled, cross-over trial found no statistically significant differences in nighttime hypoglycemic episodes between insulin aspart administered before or after meals in children and adolescents with T1D [25]. The PRONTO-T1D study in patients with type 1 diabetes showed that the rate of hypoglycemia was significantly lower for mealtime ultrarapid lispro (URLi) compared to post-meal URLi in the late postprandial period (>4 h after the meal) [26]. The data presented in this report showed higher nocturnal glucose (AUC of over 180 mg/dL) in subjects with delayed pre-dinner rapid insulin injections.

The use of second-generation rapid insulins, also known as ‘ultrarapid’ insulins, such as fast-acting insulin aspart (Fiasp^©^) and insulin ultra-rapid lispro (URLi), has been shown to improve postprandial glucose dynamics [27,28]. There is limited scientific evidence on how the type of rapid insulin injection before dinner affects the overall nighttime glucose profile, especially in people with T1D who are following an MDI regime. In the PRONTO-T1D CGM substudy, mealtime URLi decreased the nighttime TBR70 mg/dl compared with mealtime lispro [24]. However, the same study pointed out increasing glucose levels from evening to early morning in the group administered with mealtime URLi [24]. A meta-analysis of randomized controlled trials comparing faster-acting insulin aspart (Fiasp) to insulin aspart in people with diabetes mellitus showed that the nocturnal hypoglycemic episodes were not different [29].

Similarly, second-generation BI analogs, with longer, flatter, and less variable profiles, are currently available for use in the T1D population [16]. The studies comparing insulin degludec and insulin glargine 300 U/mL have shown conflicting results regarding their stability, variability, and clinical outcomes in the T1D population [17,30]. The present results indicate that, after adjusting according to the baseline glucose before dinner, using glargine U300 instead of degludec was associated with a higher TIR (70.7 vs. 58.5%, +12.2%). Our recently published study showed improved nocturnal CGM glucometrics with glargine 300 in comparison to IDeg-100 in patients with T1D in a real-world setting [18]. The present analysis supports these results.

According to a research study, a substantial number of individuals with T1D add a corrective insulin injection at least once a week, with 57% of adults and 65% of children reporting the need for it [3]. In the present study, 18.6% of the participants administered a corrective insulin dose following their evening meal. However, refraining from this practice could have a substantial impact on reducing overnight hypoglycemia and hyperglycemia, consequently leading to enhanced safety, glycemic control, and overall quality of life.

The necessity to validate and quantify the impact of insulin injection timing and type on glycemic control in real-world scenarios using CGM data, alongside the automatically recorded insulin dose and timing information, is crucial. Connected insulin pens and caps provide an opportunity to assemble this information and provide a more accurate picture of nocturnal glucose levels [9]. This study’s main strengths lie in its real-world nature and the methodology used, which includes CGM and connected insulin pen cap data. However, its limitations include the fact that the meal content was not analyzed, which could have influenced the results. Additionally, it is important to note that the detection of glucose excursions relied on CGM data obtained from sensors in interstitial fluid, rather than on direct capture in relation to the beginning of the action of ingestion. There is a delay in detecting increases in glucose levels in interstitial fluid compared to glucose levels in the blood, especially during periods of rapid change [31]. Additionally, there is a delay between food ingestion and the appearance of glucose in the bloodstream. Previous studies have indicated that both delays typically average around ten minutes each [32]. Therefore, to calculate the actual time at which intake began based on the hyperglycemic excursion data from CGM data used in the present study, at least 10–20 min should be added. The absence of analysis according to the insulin dose could be taken as a limitation. However, the study research work hypothesis starts by assuming that the dose selection is made depending on the carbohydrate counting and carbohydrate/insulin ratio and insulin sensitivity factor previously set for every subject, as per the standard of care.

## 5. Conclusions

Delayed rapid insulin injection before dinner is frequent and causes hyperglycemia overnight. A considerable number of people with T1D add a second (correction) rapid insulin injection after dinner. It significantly increases both overnight hypoglycemia and hyperglycemia. The utilization of ‘ultrarapid’ insulin has shown promise in reducing the risk of nocturnal hypoglycemia. It is recommended that second-generation ‘ultrarapid’ insulins for individuals with T1D are considered to potentially optimize glucose control. However, further research is required to determine the optimal timing of insulin injections and the impact of different insulin types on nighttime glucose levels.

## Figures and Tables

**Table 1:** Demographic and clinical characteristics and glucometrics data of study participants.

	All	Glargine U300		Degludec		*p*	URI		RI		*p*	−45/−15 min		−15/−0 min		0/+45 min		*p*
N (%)	49	16	33%	33	67%		22	45%	27	55%								
Events	775	273	35.2%	502	64.8%		306	39.5%	469	60.5%		136	17.5%	231	29.8%	408	52.6%	
TIR (%)	63.4 ± 28	70.7 ± 25		58.5 ± 30		<0.001	61.1 ± 30		61.7 ± 28		0.96	61.9 ± 29		64.2 ± 27		59.7 ± 30		0.26
TBR70 (%)	3.3 ± 11.8	3.55 ± 12		3.38 ± 12		0.81	1.7 ± 7		4.6 ± 14		0.003	4.2 ± 14		3.0 ± 11		3.4 ± 11		0.67
Low glucose events (%)	11.1	10.62		11.35		0.75	7.1		13.6		0.005	11.0		9.5		12.0		0.63
AUC70 (mg/dL × h)	119 ± 496	134 ± 526		112 ± 486		0.49	41 ± 200		171 ± 640		0.04	148 ± 540		96 ± 435		124 ± 552		0.34
AUC180 (mg/dL × h)	186 ± 256	134 ± 218		215 ± 270		<0.001	204 ± 266		175 ± 248		0.36	176 ± 271		164 ± 237		204 ± 260		0.049
Average glucose value	165 ± 42	154 ± 38		159 ± 44		<0.001	170 ± 42		162 ± 43		0.01	163 ± 44		162 ± 40		167 ± 43		0.38

AUC70, a glucose area under the curve under 70 mg/dL (3.8 mmol/L); AUC180, a glucose area under the curve over 180 mg/dL (10 mmol/L); RI, rapid insulin analogs; TBR, time below range; TIR, time in range; URI, second-generation (“ultrarapid”) analog; second-generation basal insulin (BI), insulin glargine 300 U/mL (glargine U300) and insulin degludec.

## Data Availability

The raw data supporting the conclusions in this article will be made available by the authors on request.

## Supplementary Materials

The following supporting information can be downloaded at: https://www.mdpi.com/article/10.3390/biomedicines12071600/s1, Figure S1: Rate of hypoglycemia events < 70 mg/dL; Table S1, Multivariable regression model results.
