# Supplementary material for: Nocturnal Glucose Profile According to Timing of Dinner Rapid Insulin and Basal and Rapid Insulin Type: An Insulclock® Connected Insulin Cap-Based Real-World Study
Source: Biomedicines. 2024 Jul 18;12(7):1600. doi: 10.3390/biomedicines12071600 (PMC11274448; doi:10.3390/biomedicines12071600)
Supplement: Supplementary file 1 [file biomedicines-12-01600-s001.zip › biomedicines-3031281-supplementary.pdf]

Supplementary Material

Nocturnal Glucose Profile According to Timing of Dinner Rapid Insulin and Basal and Rapid Insulin Type: An *Insulclock*® Connected Insulin Cap-Based Real-World Study

Supplementary Figure S1. Rate of hypoglycemia events < 70 mg/dL

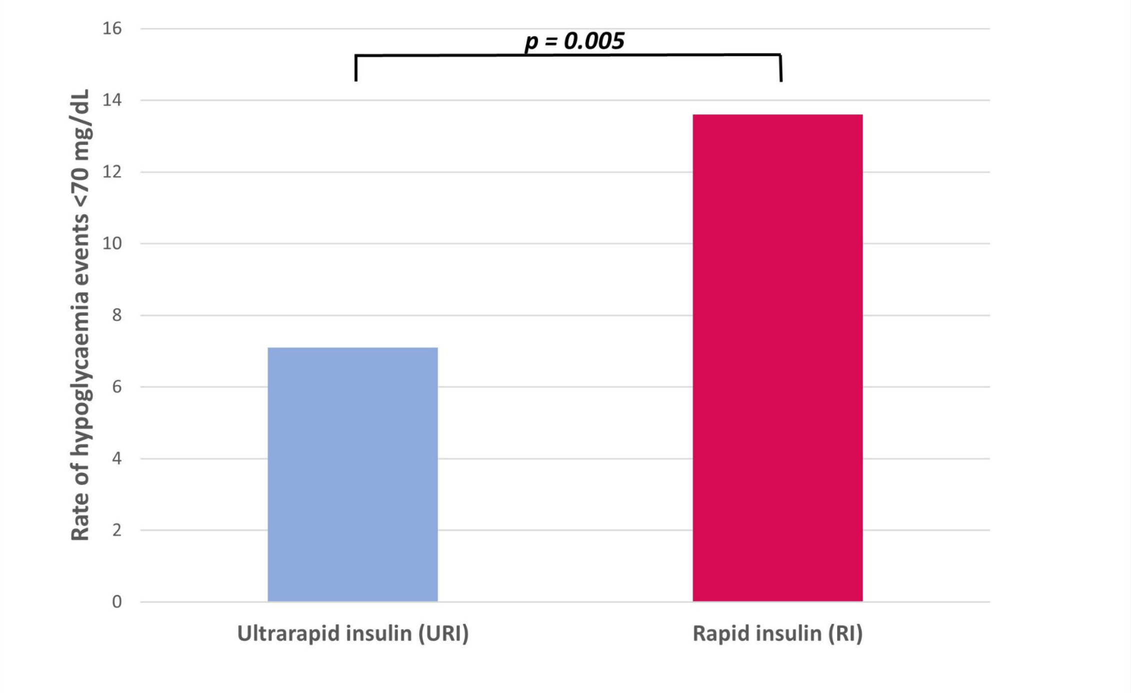

Supplementary Table S1. Multivariable regression models results: Dependent and predicting variables, adjusted R-squared, p values.

Dependant variable	Prediction models	Predictors	Adjusted R-squared	P-value
TBR	Model 1	Rapid insulin type, corrective insulin use	0,01819	0,0003088
	Model 2	Rapid insulin type, corrective insulin use, basal insulin type	0.01692	0.001052
	Model 3	Rapid insulin type, corrective insulin use. basal insulin type, rapid insulin timing	0.01604	0.003713
TIR	Model 1	Rapid insulin type, corrective insulin use	0.005807	0.0389
	Model 2	Rapid insulin type, corrective insulin use, basal insulin type	0.04972	3.39E-09
	Model 3	Rapid insulin type, corrective insulin use, basal insulin type, rapid insulin timing	0.04816	3.65E-08
TAR	Model 1	Rapid insulin type, corrective insulin use	0.00368	0.08877
	Model 2	Rapid insulin type, corrective insulin use, basal insulin type	0.04254	5.77E-08
	Model 3	Rapid insulin type, corrective insulin use, basal insulin type, rapid insulin timing	0.04027	7.00E-07
	Model 2	Rapid insulin type, corrective insulin use, basal insulin type	0.01642	0.001263
	Model 3	Rapid insulin type, corrective insulin use, basal insulin type, rapid insulin timing	0.01612	0.003622
AUC over 180	Model 1	Rapid insulin type, corrective insulin use	0.006085	0.03492
	Model 2	Rapid insulin type, corrective insulin use, basal insulin type	0.02493	5.20E-05
	Model 3	Rapid insulin type, corrective insulin use, basal insulin type, rapid insulin timing	0.02385	0.0002598
Hypoglycemic events	Model 1	Rapid insulin type, corrective insulin use	0.01836	0.0002886
	Model 2	Rapid insulin type, corrective insulin use, basal insulin type	0.01724	0.0009318
	Model 3	Rapid insulin type, corrective insulin use, basal insulin type, rapid insulin timing	0.01676	0.002928

AUC, glucose area under curve (AUC) over 180 mg/dL (10 mmol/L); Hypoglycemic events, the rate of overnight periods (%) with hypoglycemic events defined as periods of glucose levels under 70 mg/dL (3.9 mmol/L) lasting more than 15 minutes; TAR, time over 180 mg/dL (10 mmol/L); TBR, time below the range of glucose 70 mg/dL (3.9 mmol/L); TIR, time in range 70–180 mg/dL (3.9–10.0 mmol/L).
